# Sleep Disturbances in Newborns

**DOI:** 10.3390/children4100090

**Published:** 2017-10-20

**Authors:** Daphna Yasova Barbeau, Michael D. Weiss

**Affiliations:** Department of Pediatrics, University of Florida, Gainesville, FL 32610, USA; mweiss@ufl.edu

**Keywords:** sleep, sleep disturbances, premature neonate, sleep wake cycling, sleep states, sleep disorder, apnea, aEEG

## Abstract

The purpose of this review is to serve as an introduction to understanding sleep in the fetus, the preterm neonate and the term neonate. Sleep appears to have numerous important roles, particularly in the consolidation of new information. The sleep cycle changes over time, neonates spend the most time in active sleep and have a progressive shortening of active sleep and lengthening of quiet sleep. Additionally, the sleep cycle is disrupted by many things including disease state and environment, and the amplitude integrated EEG can be a useful tool in evaluating sleep, and sleep disturbances, in neonates. Finally, there are protective factors for infant sleep that are still being studied.

## 1. Introduction

Sleep is a necessary function of life. Studies consistently show the importance of sleep in its role for cognitive function and developing memories. Most importantly, sleep solidifies new information [1]. Sleep deprivation in adults leads to ineffective attention, learning, emotional regulation, and decision-making. Neonates and children show a similar response. Inadequate sleep in children has been associated with increased adiposity, poor emotional regulation, poor overall well-being, and decreased academic performance [2]. Because fetuses and neonates spend most of their day asleep, adequate sleep is particularly important for them [3].

This chapter will focus on sleep during the neonatal period. The chapter will examine the development of sleep in the fetus, and the preterm and term neonate. A summary of the developmental changes, as seen on amplitude integrated electroencephalogram (aEEG) and EEG, from the preterm to the term neonate will provide a foundation for the bedside clinician. This should serve as a broad introduction to the alterations in sleep that are encountered in the Neonatal Intensive Care Unit (NICU) and how they appear in conjunction with a variety of pathophysiologic entities. Furthermore, clinicians will learn how sleep alterations can serve as a marker of long-term outcomes in neonates requiring intensive care.

## 2. Development of Sleep

### 2.1. Sleep States

“Sleep” is composed of various stages or categories (Table 1). The first category, quiet sleep, is characterized by closed eyes, no eye movements, decreased body movements, slow waves on EEG, and regular respiration. The second category is active sleep, which includes rapid eye movement (REM) sleep. An infant in active sleep typically has closed eyes, eye movements, body and limb movements, low voltage EEGs, and irregular respirations. It is possible to be in active sleep without REM [1]. Notably, a lack of REM sleep in neonatal rats leads to sleep and behavioral problems and smaller cortical size [4]. The third category is indeterminate or transitional sleep, which shows components of both quiet and active sleep. Finally, waking is characterized by open eyes, irregular and active respirations, as well as eye and body movements. Progression through these cycles changes with advancing post-natal age. For example, the duration of cycles decreases during the day time as infants age [5].

Studies have shown that sleep changes developmentally. These changes to sleep include lengthening of the sleep interval, increased quiet sleep, and decreasing REM or active sleep. Additionally, the amount of time spent in transitional sleep decreases with age [6,7,8]. The following sections will examine sleep development in the fetus, the preterm neonate, and the term neonate.

### 2.2. Fetal Sleep

Most data on fetal sleep were obtained from animal models such as sheep and primates, often baboons. Fetal sleep states are not present in primates until the second half of gestation [13]. Movements in the fetus begin as early as 7–8 weeks postmenstrual age and distinct patterns of motion emerge at approximately 15 weeks postmenstrual age [14]. Body movements and “breathing” movements occur in both quiet sleep and active sleep. These movements are important in strengthening the fetal muscles and preparing for breathing postnatally because the lungs are not used for oxygenation while in the womb. Researchers do not think that wakefulness is very active in utero because many components of the environment suppress it. These components include warmth and various chemicals secreted by the placenta and fetal brain that are present in fetal blood (e.g., prostaglandin E2, adenosine, pregnanolone, and allopregnanolone). However, fetuses do appear to have periods of more vigorous activity that includes an accelerated heart rate and swallowing and even fetuses as young as 20–28 weeks appear to have these distinct rest and activity patterns [15]. The cyclical rhythm of activity is likely guided by placental transfer of maternal melatonin [16].

### 2.3. Sleep in Premature Neonates

**Figure 1:**
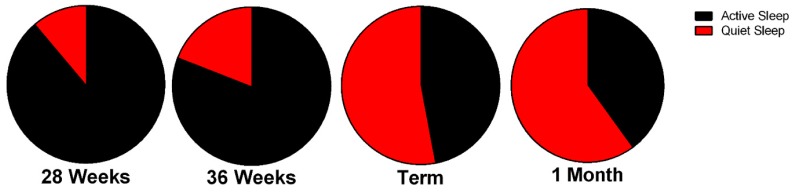
Active versus quiet sleep in neonates at 28 weeks, 36 weeks, term, and 1 month. The duration of quiet sleep increases as the neonate matures. The figure is adapted from [24].

Similar to fetal life, premature neonates have delineated periods of active and quiet sleep that can be detected as early as 25–27 weeks gestational age. Premature neonates can spend up to 90% of their time sleeping, which decreases as they age [17]. In comparison, the full-term infant sleeps approximately 70% of the time [16,18]. The sleep progression continues as the preterm neonate ages. The amount of transitional sleep decreases, and more discrete periods of sleep and wakefulness occur (Figure 1). Up to 80% of sleep in the preterm neonate is marked by periods of active sleep. This amount decreases as the neonate ages [19]. Notably, within the active cycle, true REM behavior also increases over time and active sleep without REM sleep decreases over time. This indicates better organization of the active component of the sleep cycle over time [20]. In adults, active sleep is synonymous with REM sleep [21]. This REM-type sleep is particularly important. Active sleep with low REM behavior is found in groups of neonates with developmental delays. For example, in a cohort of premature neonates, the babies with higher medical risk scores tended to have less REM activity during active sleep. In this cohort, those neonates who spent less time in REM sleep spent a higher percentage of time crying or fussing or were generally characterized as “unfocused” [22]. By 35 weeks gestational age, indeterminate sleep decreases to less than 10% of the total sleep-wake cycle [19]. The average late preterm (>35 weeks gestational age) neonate’s sleep-wake cycle was approximately 50–60 min, as measured by continuous polysomnography [23].

At term-corrected age, most premature neonates have sleep-wake states similar to those of typical full-term newborns over the course of the first year of life [7,19]. In fact, very preterm neonates may mature their sleep-wake cycles sooner than term neonates [24]. However, when compared to their full-term counterparts, premature neonates have longer sleep duration both during the day and at night and more nighttime awakenings throughout the first year of life [7,25]. Additionally, preterm neonates are much more likely to initiate the sleep cycle in active sleep, meaning that they have longer quiet sleep latency. In addition, their overall sleep latency, the time it takes to fall asleep, is variable across the first year of life. Over the course of the first year, quiet sleep becomes predominant in the first third of the night, as compared to the last third of the night, where active sleep preponderates [7]. In a study of neonates, caregivers of preterm neonates more frequently said that their infants had sleeping problems and were noisy breathers while sleeping, as compared to caregivers of full term neonates. This study also confirmed that even at six months, preterm neonates had greater apnea-hypopnea indices. For example, greater than 80% of premature neonates had more than one event per hour, confirming their caregivers’ observations [25].

Preliminary studies show that infants without mature sleep-wake cycling by 34 weeks gestational age have lower scores on neurodevelopmental indices at nine months, but these correlations have not persisted at 18 months [26]. Premature babies with less REM sleep, regardless of the time spent in active sleep, in particular, performed poorly on an infant’s mental development index, using the Bayley II developmental screening tool, than infants who spent more time in REM sleep during active sleep [22]. Poor neonatal sleep also predicted longer gaze duration during a new visual recognition memory task and increased distractibility at 18 months [27]. However, the few longitudinal studies available failed to find a difference in sleep behaviors in preterm infants when compared to term infants long-term [9].

### 2.4. Sleep in Term Neonates

Full-term infants spend approximately half of their overall sleeping time in each of the sleep states: quiet sleep and active sleep [16,18,19]. Sleep-wake cycles progress as a function of post-menstrual age. As infants age, their sleep-wake cycles shorten, and their daytime cycles become shorter than their nighttime cycles [5,8]. As described in the preterm neonate, active sleep decreases over the first year of life, and quiet sleep increases over that time. Similarly, quiet sleep becomes more predominant early in the night and more active sleep becomes more predominant later in the night, during the longest sleep period. In contrast to preterm infants who have a slow decline in active sleep over the first year, full term infants have a stable active sleep of about 50% until nine months of age when a steep decline to 40% is evident. By one year of life, term and preterm neonates corrected for gestational age spend approximately 40% in active sleep and approximately 45% in quiet sleep. Both groups spend an increasing time awake over the first year of life. Unlike age-corrected preterm neonates, full-term neonates have gradually decreasing sleep onset latency; over time they fall asleep sooner. For both groups, sustained sleep periods increase from approximately four hours at two weeks, to approximately seven hours by five months, and this longer sleep period shifts to the night time [7].

## 3. Bedside Tools to Monitor Sleep

Many tools are available to evaluate sleep in infants, though the most reliable methods are the most intrusive methods. To date, a reliable method that does not require expertise to interpret and that can be used long-term without damage to infant skin or significant technical support is not available. Newer sleep detecting technology is in development but remains experimental (please see [12] for an in-depth review of the topic).

### 3.1. Behavioral Classification

Awareness of a neonate’s motor movements, specific body movements (especially the opening and closing of the eyes), as well as a neonate’s breathing pattern can provide a useful classification for sleep (Table 1). Active sleep is characterized by closed eyes, with eye movements noted beneath the eyelids, bursts of sucking, and small muscle twitches with low muscle tone between startles. Neonates in quiet sleep have closed eyes as well, with episodes of rhythmic mouth movements, and little to no motor activity. Neonates who are awake have open eyes with distinct, higher-intensity motor movements [12]. While these classifications provide reasonable rough estimates for the neonatal sleep state, clinical observation should accompany all electronic monitoring, behavioral monitoring alone is not entirely accurate because many of the sleep states have overlapping characteristics. One small study showed that agreement between observers of behavioral states was less reliable than the agreement between observers using EEG patterns (77% versus 87%, respectively) [28].

### 3.2. Heart Rate Variability (HRV)

In addition to behavioral monitoring, heart rate variability (HRV, the measurement of intervals between heartbeats) may be a marker for neonatal sleep. HRV reflects the rapid and dynamic changes in autonomic regulation caused by the interplay of the sympathetic and parasympathetic nervous systems. HRV serves as a biomarker for numerous disease states in both neonates and adults. Neural inputs from both branches of the autonomic nervous system regulate this beat-to-beat variability. The influence of the separate branches can be quantified using a frequency-based or spectral analysis of the heart periods [29]. HRV increases as an infant develops. Importantly, in terms of sleep-wake cycling, HRV depends on the sleep state. Overall, heart rate decreases with quiet sleep [10,30]. Preliminary studies have shown that HRV may be sensitive enough to distinguish the sleep states in preterm infants greater than 30 weeks post menstrual age [31]. While HRV is potentially useful, it requires software translation of output and is not currently used in routine bedside monitoring.

### 3.3. Polysomnography

Polysomnography is the gold standard for monitoring sleep, however it requires a variety of measurements and sensors, which make it less useful in clinical practice (please see [32] for an in depth review of indications for clinical usage). Polysomnography requires pulse oximetry, EEG leads, electrooculography (EOG), chin electromyography, and electrocardiogram (ECG), or other heart rate monitoring and pulse oximetry [33]. Polysomnography has the added benefit of providing the timing and frequency of infant apnea, or brief cessations in breathing. It also has the potential to indicate whether these apneas are central (originating from the brain, common in immature brains or any patient with brain injury) or obstructive (either from relaxation of the muscle in the throat, or from patient positioning) [32].

### 3.4. EEG

EEG provides information about brain function and activity over time that allows clinicians to observe maturation of the central nervous system. In particular, EEG observes changes in brain wave activity during sleep with maturation, thus providing a tool for diagnosing brain disorders and a prognosis with injury (please see [34] for an in-depth review of sleep-EEG in preterm and term neonates).

In neonates less than 30 weeks of gestation, a clear differentiation between wake and sleep states is often impossible to discern on the EEG [34,35]. Because an EEG alternates between periods with and without measurable cerebral activity, sleep states may not be discernible, even though preterm neonates between 25–30 weeks do appear to cycle between all of the sleep states. Inter reliability of scoring EEGs tends to improve as the infant gestational age increases [34]. In fact, the American Academy of Sleep Medicine recommends only scoring the EEG by two patterns: continuous and discontinuous in infants zero to two months of age, rather than by specific sleep state [34]. The finding of active sleep on EEG is marked by irregular delta activity and has amplitudes up to 300 µV, and will demonstrate epochs of REM. The second pattern of low amplitude activity is termed discontinuity. After 30 weeks of gestation, the percentage of time that a neonate spends in discontinuity decreases as gestational age increases [35,36]. Between 30 and 31 weeks, there is increasingly recognizable patterns of quiet and active sleep [34]. The background pattern for the term neonate, described below, develops between a gestational age of 32 and 37 weeks [35]. For most infants, there is a reliable rhythmicity of sleep wake cycling by 36 weeks [37].

**Figure 2:**
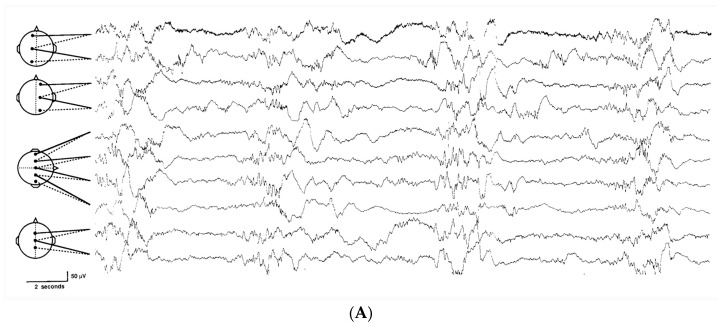

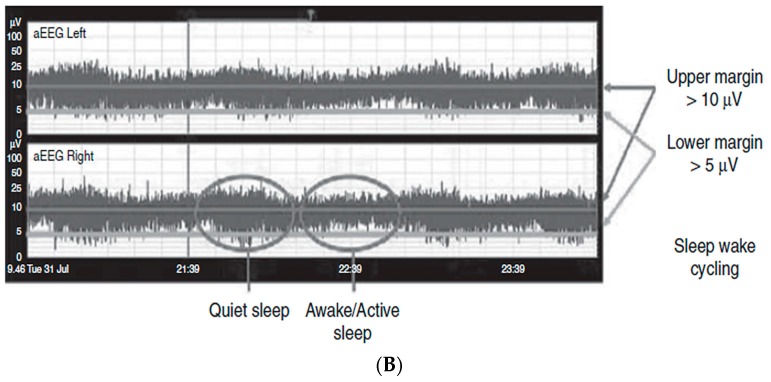
Trace alternant in a term baby (**A**) and sleep-wake cycling in an amplitude integrated EEG (aEEG) (**B**). Note the alternating background in (**A**). (**B**) demonstrates expansion of the background pattern during quiet sleep and narrowing during awake/active sleep. Quiet sleep on the aEEG is the equivalent of trace alternant on the EEG. Figures are derived from [32,38].

The EEG in a full-term neonate in active (REM) sleep shows a background pattern of continuous activity. This is demonstrated over all regions without variations in amplitudes up to 70 µV. Quiet sleep (non-REM) is characterized by an alternating background pattern with 3 to 10 second bursts of theta and delta activity (amplitudes up to 200 µV) intermingled with periods of fast activity in alpha and beta frequencies, as well as some isolated theta waves with amplitudes between 50 and 70 µV [35]. This pattern during quiet sleep is referred to as “tracé alternant” (Figure 2A) after its characteristic changes from high- to low-amplitude activity [35].

### 3.5. Amplitude Integrated EEG (aEEG)

The amplitude integrated EEG (aEEG) continuously monitors brain function and can be easily interpreted by bedside clinicians in real time. The original aEEG device was referred to as the cerebral function monitor and was created by Prior and Maynord in the 1960s for use in the adult intensive care unit [38]. The device was applied to neonates in the late 1970s and early 1980s [39,40]. The concept has been refined over the years, and current devices display the aEEG trace along with the raw EEG.

**Figure 3:**
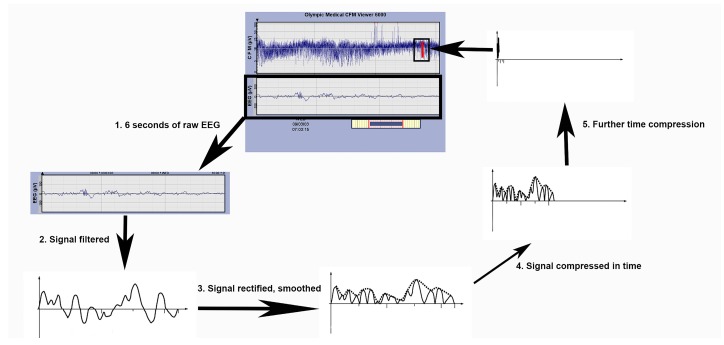
The aEEG derives a tracing from the raw EEG. In step 1, six seconds of the raw EEG is extrapolated. In step 2, the signal is filtered. The filtering includes an asymmetric band pass filter that attenuates activity below 2 Hz and above 15 Hz. In step 3, the signal is rectified and smoothed. Note the negative inflections are converted to positive inflections. In steps 4, 5, and 6 the signal undergoes a series of time compressions. The time compressions steps then lead to a single line. Multiple lines then form a pattern for interpretation. The amplitude display on the bedside monitor is linear between 0 to 10 µV and logarithmic between 10 to 100 µV (Derived from [29]).

The aEEG tracing is derived from a limited number of channels of the conventional EEG, which uses the 10–20 international system of electrode placement, where odd numbers are placed to the left and even numbers on the right [41]. The aEEG typically employs biparietal leads (channels P3 and P4 on the conventional EEG montage), which correlate to each of the neonate’s parietal lobes and leads on each side of the head (the “central” channels C3 and C4 on the conventional EEG montage). While there is no “central” lobe, these lead locations sit in the space between the frontal and parietal lobe channels in the 10–20 system. Interestingly, much activity in neonates comes from the central leads and this is why they were included [41,42]. The raw EEG is both time compressed and filtered before display (Figure 3) [41,42]. Specifically, the filtering includes the following: an asymmetric band pass filter that attenuates activity below 2 Hz and above 15 Hz, semi-logarithmic amplitude compression, rectifying and smoothing, and time compression. Once this filtering step occurs, the raw EEG of six seconds is converted into a single line on the display. The lines eventually form a trace, which can be interpreted employing basic pattern recognition. The bandwidth of the trace on the display of the monitor reflects the minimum and maximum EEG amplitude. The amplitude display on the bedside monitor is linear between 0 to 10 µV and logarithmic between 10 to 100 µV. This semi-logarithmic display enhances detection of changes in the low-voltage activity and avoids overloading the display at high amplitudes [38]. The electrode impedance is recorded to ensure the technical quality of the tracing.

The bedside clinician can use the aEEG to detect the presence of sleep-wake cycling (SWC). SWC is characterized by regular variations in the aEEG bandwidth. Typically, a narrow bandwidth corresponds to wakefulness or active sleep. A wide bandwidth denotes quiet sleep [43].Cyclic variations in the aEEG background suggestive of immature SWC are seen in healthy preterm neonates around 26 to 27 weeks of gestation. SWC develops with increasing maturation from 31 to 32 weeks of gestation. At the same time, quiet sleep periods are easily discernible in the aEEG as periods that have increased bandwidth [38]. At term, the more discontinuous aEEG during sleep represents “tracé alternant” in the EEG [38].

## 4. Alterations of Sleep in the NICU

Premature neonates are at significant risk for abnormal brain development. These risks are compounded in neonates that require intensive care in a Neonatal Intensive Care Unit (NICU). These infants have impaired sleep patterns for a variety of reasons, including certain underlying disease processes, frequent interventions, abnormal day-night cycles, around-the-clock lighting, and frequent, chaotic ambient noise. A growing body of literature shows that sleep disruption by even routine handling of preterm neonates in the NICU is further affecting their brain maturation process [23].

In a study of SWC that monitored term neonates who were at risk for neurologic abnormalities but were medically stable and felt to be at low risk for respiratory decompensation, researchers found that neonates spent 27%, or approximately 65 min, out of a 4-hour recording with provider contact. While some interactions were related to technical support of the study equipment, numerous interactions with the infant or devices within the isolette still occurred in a 4-hour period. During these observations only 50% of the infants were able to engage in a full sleep-wake cycle before being disturbed for care. Notably, 57% of the infant-provider contacts resulted in full awakenings, or at least arousal. Many of these events were accompanied by hypopnea, apnea, or desaturation. The infants appeared particularly susceptible to desaturation when awake and during the indeterminate sleep state, but were particularly vulnerable to hypopnea and apnea during active sleep. Neonates in active sleep may be difficult to distinguish from awake infants since neonatal movements and postures are similar. The only difference between the sleep states is that the eyes may be open when awake and closed in active sleep [23].

Neonates in intensive care may also be impacted by different types of breathing support. For example, infants less than 32-weeks’ gestation on heated humidified high flow nasal cannula had increased activity and spent less time asleep when compared to infants requiring continuous positive airway pressure (CPAP) ventilation, despite CPAP being described as increased respiratory support [11]. Because ventilated neonates have a higher acuity, a comparison of their sleep to controls is difficult.

Disordered sleep accompanies many disease processes in the NICU (Table 2). Researchers do not know if the sleep disturbances encountered in these patients contribute to long-term deficits or merely serve as a biomarker for these deficits. In the following section, several pathophysiologic entities encountered in the NICU will be reviewed. Disturbances of sleep in neonates are probably encountered in other disease processes as well but have not been studied or have only been studied in small pilot projects.

### 4.1. Chronic Lung Disease (CLD)

Chronic lung disease (CLD) affects infants that require prolonged ventilator or oxygen support, and is common in prematurity but also occurs in a variety of other conditions. CLD is accompanied by significant respiratory morbidity and is also independently associated with post discharge mortality [44,45]. Sleep appears to be a particularly vulnerable time for neonates with CLD. Even neonates with CLD who have normal oxygen saturations while awake appear to suffer from periods of hypoxia (less than 90% oxygen saturation but as low as less than 80%) while asleep [39]. These hypoxic episodes may not be identified clinically because they are frequently unrelated to apnea, bradycardia or cyanosis, and because oximetry measurements may only be done when awake. In fact, a similar study from another group of researchers of infants with CLD identified that awake oxygen saturation did not correlate with asleep oxygen saturation [46]. A study of infants and children less than three with CLD found that they had a higher mean respiratory disturbance index, which reports the number of respiratory events during sleep. These events include respiratory effort-related arousals that disrupt sleep, regardless of sleep state. Fortunately, the respiratory disturbance index decreased with age, even in patients with chronic lung disease [47]. Unfortunately, infants with even mild desaturation to 88–91% for >1 hour while asleep showed a decreased average daily weight gain when supplemental oxygen was discontinued as compared to infants who maintained saturations of >92% while asleep [46]. This highlights the point that oxygen saturation should be monitored in these infants, particularly while asleep [32].

### 4.2. Hypoxic-Ischemic Encephalopathy

Hypoxic-ischemic encephalopathy (HIE) is a significant cause of morbidity and mortality in neonates. The incidence of HIE ranges from 1 to 8 per 1000 live births in developed countries to as high as 26 per 1000 live births in underdeveloped countries [62]. Up to 60% of asphyxiated newborn infants who exhibit HIE die during the newborn period. Among the survivors, 25% or more show permanent neuropsychological handicaps in the form of cerebral palsy (CP) with or without associated mental retardation, learning disability, or epilepsy [63]. Whole-body hypothermia reduces the risk of death or disability in infants with moderate or severe HIE [64,65,66].

HIE disturbs sleep organization [67]. SWC is defined as a state of continuous normal voltage and the presence of both wakefulness or active sleep and quiet sleep with a minimum of two or three consecutive sleep state changes on aEEG for a duration of 20 min during a 3- to 4-hour period [48]. In neonates with mild HIE not undergoing hypothermia, SWC, as detected on the aEEG, occurs at a median of seven hours of age. Neonates with moderate and severe HIE do not have the occurrence of SWC, as measured by an aEEG, until a median of 33 (moderate) and 62 (severe) h of age [67]. The return of SWC correlates with developmental outcomes. Neonates with HIE who developed SWC on the aEEG within 36 h of birth had a higher median Griffith’s developmental quotient at 12–66 months of age than those who showed SWC at a later stage [67]. In addition, sleep architecture is disturbed with HIE. Neonates with evidence of birth asphyxia spent 18.9% of their sleep in active sleep as compared to 44.7% in a non-asphyxiated control group, as measured by EEG. In addition, asphyxiated neonates had an increased percentage of quiet sleep (46.5%) when compared to the non-asphyxiated controls (38.7%) [49].

Hypothermia has become the standard of care for neonates with HIE. Hypothermia further affects the return of SWC. Thoresen, et al. (2010) examined the effect of hypothermia on the aEEG of neonates randomized to hypothermia as part of the hypothermia clinical trials and compared them to neonates randomized to normothermia as controls [50]. Neonates who underwent hypothermia and normothermia were further classified as having a good or poor neurodevelopmental outcome based on the results of a Bayley Scales of Infant Development test (Bayley II). The median time of the onset of SWC in neonates with good outcomes was 24 h in normothermia treated neonates and 36 h in hypothermia treated infants [50]. Never developing SWC strongly predicted death and disability. The odds ratio for a poor outcome increased by 1.05 for every 1-hour delay in achieving SWC in neonates undergoing hypothermia [50].

Long-term effects of neonatal HIE such as CP can also affect sleep. The sleep dysregulation in infants with CP is multifactorial and significant. As compared to their peers, children with CP are seven to twelve times more likely to suffer from sleep disorders and other comorbidities that impact sleep. For example, seizure disorder, behavioral issues, visual impairment (which impacts circadian rhythm), abnormal hearing, and disordered breathing all play a role in abnormal sleep architecture for children with CP. Children and adolescents with CP self-report higher fatigue, increased anxiety, and increased stress levels, which additionally impact poor sleep [68].

In summary, HIE disturbs SWC and the quality of sleep in neonates and may serve as a marker for the bedside clinician to judge the degree of injury and predict long-term outcomes. Long-term neonates who suffered from HIE may have ongoing sleep disruption.

### 4.3. Congenital Heart Disease

In normal term neonates, SWC normally emerges within the first 12 h of life and can be present immediately after birth [69,70]. Using aEEG, SWC in neonates with congenital heart disease was present in only 50% by 24 h of age and delayed beyond 72 h in 48% [51,52]. Sleep wake-cycling was found to occur more frequently in neonates with coarctation of the aorta [51]. Notably, neonates with congenital heart disease are at risk for brain injury and/or hypoxic-ischemic injury, which may account for the delay in SWC [51].

### 4.4. Neonatal Abstinence Syndrome (NAS)

Neonatal abstinence syndrome (NAS) is a clinical diagnosis, and a consequence of the abrupt discontinuation of chronic fetal exposure to substances that the mother used or abused during pregnancy [71].

Prenatal exposure of the fetus to selective serotonin reuptake inhibitors (SSRI) can affect sleep in the neonate. Analysis of the sleep patterns showed that neonates exposed to SSRI had more REM sleep than non-exposed neonates. During a 15-minute continuous sleep period, SSRI-exposed neonates had an increase in motor activity related to startles during REM sleep. Differences were not observed between exposed and non-exposed neonates for quiet sleep [72].

Maternal opiate use has increased from 1.2 mothers per 1000 live births in 2000, to 5.6 mothers per 1000 live births in 2009. Corresponding to the increase in maternal usage over the same time span, the number of neonates diagnosed with NAS has increased from 1.2 to 3.4 per 1000 live births [73]. The number of neonates who had NAS and were admitted to the NICU increased by 10-fold from 2005 to 2011 in the State of Florida [74]. NAS symptoms in this patient population include multiple signs and symptoms that involve multiple systems. Central nervous signs include irritability, jitteriness, tremors, and excessive crying. The hyperirritability can lead to agitation, difficulty sleeping, and inconsolable crying [71]. With respect to sleep, newborns withdrawing from opiates have a significant increase in active sleep with a reciprocal decrease in quiet sleep as compared with controls [53,55]. Neonates who are symptomatic and undergoing treatment for NAS have an increase in sleep fragmentation with more than 50% of arousals progressing to wakefulness. These data suggest that neonates with NAS seem to have a lower threshold for wakefulness associated with an arousal stimulation compared to normal healthy neonates [54].

### 4.5. Inborn Errors of Metabolism

Inborn errors of metabolism are rare congenital disorders of metabolism, in which an enzyme that facilitates conversion of substrates to products are defective and cause a block in key metabolic pathways. Neonates with inborn errors of metabolism typically present with encephalopathy, seizures, poor feeding, respiratory disturbances, and recurrent vomiting [56]. The presenting symptoms are dependent on the blocked metabolic pathway. An international registry of inborn errors of metabolism has reported disturbances in both the background pattern and SWC [56]. Inborn errors of metabolism associated with an absence of SWC include long-chain keto-thiolase deficiency, pyruvate dehydrogenase deficiency, urea cycle defects, maple syrup urine disease, and non-ketotic hyperglycinemia.

## 5. Protecting Infant Sleep

Researchers have studied the effect on sleep of many interventions that target development (Table 2). Unfortunately, nearly all of these studies focus on short-term benefit and most do not discuss the long-term benefits to development. In addition, many of these studies have small sample sizes and are not blinded. However, developmental interventions that improve infant sleep could improve overall long-term neurodevelopmental outcomes. More research on the topics below can help shape the artificial environment of the NICU. The most-studied intervention, Kangaroo Care, is described first.

### 5.1. Kangaroo Care (KC)

Kangaroo care (KC), or holding an infant in skin-to-skin contact with a parent, is associated with many benefits for the mother-infant dyad. Decreased hypothermia, decreased hypoglycemia, and increased exclusive breastfeeding rates are associated with KC for low birth weight infants. Infants undergoing kangaroo care had decreased respiratory rates and higher oxygen saturation rates, as well as lower HRV associated with fewer episodes of extreme bradycardia (common in premature neonates) and both mothers and infants have lower cortisol during episodes of KC [57,75,76]. Increased KC is associated with improved growth velocity and increased head circumference growth in preterm infants [64]. Finally, studies have demonstrated decreased overall infant mortality in infants that have undergone KC.

Recent studies showed that infants who undergo KC also have better sleep. KC accelerates neurobehavioral maturation of sleep. Infants who did KC with their mothers for as little as 14 days, for 1 hour per day, had more organized sleep states and spent more time in both active and quiet sleep at term corrected age [57,76]. Infants undergoing KC have more mature SWC and demonstrate fewer arousals from sleep [58].

### 5.2. Infant Massage

Premature infant massage is increasingly being studied. While it does appear to confer some benefit to infants receiving it, massage does not significantly alter long-term neurodevelopmental outcomes and is time consuming. Massage does decrease infant crying, decrease infant pain scores, improve infant weight gain in preterm and low birth weight infants, and potentially decreases their length of stay [59,77,78]. Some studies indicate that infant massage improves mother-infant interactions after the massage intervention [59]. Additionally, studies of infant massage and gentle human touch found a significant increase in sleep state and a decrease in awake state, both during and after the intervention [3]. Fortunately, studies have not found negative effects, and certain types of infant massage can be easily taught to parents, helping engage them in the care of their infants [78].

### 5.3. Light Modification

Modifying the NICU environment by changing lighting makes some difference to infants. Either cycled light (creating day and night light patterns) or maintaining near darkness has benefit. In particular, cycled light, as compared to continuous bright light benefits preterm infants. Studies show a decreased length of stay, a trend towards fewer ventilator days, and a shorter time to full feeds [79]. Cycled lighting long term, over 20 to 30 days, changed infants’ day and night activity as compared to controls [3]. Additionally, preterm infants exposed to cycled light slept longer in the nighttime than infants who had not been exposed to day-night light cycling [24].

### 5.4. Sound Modification

The NICU can be a very noisy place, even for infants within an incubator. In fact, infants have awakenings during sleep that are caused by even brief sound peaks [80]. This excessive stimulation is known to cause apnea and fluctuations in other vital signs. Prolonged exposure to noise puts premature infants at an additional risk for hearing deficits, which can translate into difficulties with speech and language [81]. Interestingly, an increasing number of studies are targeting music therapy in preterm infants. Music, particularly live music, in low birth weight infants early in the stay improved infant sleep, heart rate stability, suckling, and feeding [60,61].

## 6. Future Directions for Research

While much progress has been made to understanding fetal and infant sleep patterns, there is still much we do not understand about both the short and long-term impact of sleep and of the impact of interventions on sleep (Table 3).

## 7. Conclusions

This chapter outlined the development of sleep from the fetus to the term neonate. Bedside tools available to the clinician have increased our understanding of how the neonatal intensive care impacts sleep. An important remaining question is if the disturbances of sleep are merely a biomarker for long-term outcomes, or a point at which interventions can be performed to improve outcomes. Unfortunately, clinicians still do not know which interventions would be of most benefit.

## Figures and Tables

**Table 1:** Summary of Sleep States. Figures derived from [5,7,9,10,11,12]

	Eyes	Body Movements	Facial Movements	Respirations	EEG Findings	aEEG Findings ^a^
Quiet Sleep	Closed	Relatively no body movement	Few rhythmic mouth movements	Regular	Trace alternant (medium to high voltage) 30–200 µV	Wide bandwidth
	No eye movements		Relaxed Sighs	Slow		
Active Sleep (AS)	Closed	Slow, small twitches	Frowns and smiles	Irregular	Continuous (low voltage) 30–70 µV	Narrow bandwidth
	Rapid eye movements	Low tone between startles	Burst of sucking Some vocalizations			
Transitional Sleep	Periods of opening and closing	Slow startles	Grimace, intermittent sucking	Regular	Continuous (high voltage) 100–200 µV	Variable
	Slow eye movements		Increase in vocalizations			
Awake	Open	Rapid startles	Frowns, smiles, grimace, sucks, crying	Irregular	Continuous (medium voltage) 70–100 µV	Narrow bandwidth
	Rapid or slow eye movements	Gross motor movements	Vocalization			

^a^ Movement between sleep states produces a sinusoidal pattern.

**Table 2:** Neonatal disorders associated with sleep disruption.

	Alteration/Protective	Overview	How Sleep Is Affected	Comments	References
**Hypoxic-Ischemic Encephalopathy**	Alteration	Brief disruption of blood flow to the infant brain can have a profound effect on neurodevelopment.	Delayed SWC-progressive with worse injury	Infants undergoing cooling who developed SWC by 36 h of age had better outcomes	[48,49,50]
			Decreased AS		
**Congenital Heart Disease**	Alteration	Many anatomic cardiac defects produce hypoxemia	Delayed SWC for age	May be accompanied by HIE, prolonged hospitalization and surgery, which also complicate sleep	[51,52]
**Neonatal Abstinence Syndrome** **(NAS)**	Alteration	Prenatal illicit or prescription drug exposure is becoming an increasing concern	SSRI: More AS and increased motor activity during AS		[53,54,55]
			Opiate: more AS, low threshold for arousal, fragmented sleep		
**Inborn Errors of Metabolism**	Alteration	A family of disorders caused by enzyme deficiencies in the metabolic pathways	Spectrum of pathology from no cycling to normal cycling depending on enzyme deficiency	Encephalopathy itself alters the aEEG	[56]
**Chronic Lung Disease**	Alteration	Babies with prolonged oxygen needs	Lower saturations while asleep	Infants on oxygen should be evaluated while asleep before discontinuation	[32,46,47]
			Increased respiratory events and arousals during sleep		
**Kangaroo Care**	Protective	Placing an infant skin to skin with a parent provides warmth and familiar stimulation	More mature SWC		[57,58]
			More time asleep Fewer arousals		
**Infant Massage**	Protective	Various techniques: firm pressure, gentle stroking, containment	Increased sleep state and decreased awake state		[3,59]
**Light Modification**	Protective	Intensive care unit is inherently bright	Cycled light long term associated with differences in day night activity		[3,24]
		Options are to provide day/light patterns for light or to reduce light	Longer night time sleeping		
**Sound Modification**	Protective	Intensive care unit is inherently noisy	Episodes of noise can cause arousals	Live music, either mother singing or instruments in the unit, associated with best outcomes	[60,61]
		Can decrease noxious noise or increase pleasant noise	music improved infant sleep		

**Table 3:** Research questions answered and research questions that remain, regarding neonatal sleep.

Age	Questions Answered	Questions Remaining
**Fetus**	Fetuses do appear to have cyclical behavioral patterns [13,14,15,16]	Does maternal activity significantly impact fetal sleep wake behaviors? Do maternal and fetal conditions/pathology affect fetal sleep wake behaviors? Do fetal patterns by gestational age match their premature counterparts?
**Preterm Infant**	Preterm infants do have predictable sleep development [7,17,20,24,26,36,82] REM sleep is particularly important in development [4,22] Routine care impacts sleep in the NICU [11,23,25,83] EEG and HRV can be used to follow infant sleep progression and estimate infant sleep stage [12,30,34,42,75] Disease of premature infants do impact sleep [32,46,47] Interventions in the NICU can help improve infant sleep [3,75,84,85]	Do preterm infants with poor sleep have worse outcomes than preterm infants with better sleep? What effect do interventions in the NICU have on long term development? Can monitoring of preterm sleep performed in a manner that is noninvasive and unobtrusive over time? Can monitoring of preterm sleep direct goals of care? Do diseases of prematurity that dysregulate sleep impact long-term outcomes?
**Term Infant**	Full term infants have predictable sleep development [7,16,18] REM sleep is particularly important in development [4,6] Sleep dysregulation can impact school aged outcomes [2]	Do sleep interventions in the home affect long-term outcomes? Do different methods of infant “sleep training” effect infant sleep and long term outcomes? What are the long-term effects of sleep dysregulation in infants with HIE and NAS?
